# The Psychological Impact of COVID-19 in Italy: Worry Leads to Protective Behavior, but at the Cost of Anxiety

**DOI:** 10.3389/fpsyg.2020.566659

**Published:** 2020-12-10

**Authors:** Giulia Prete, Lilybeth Fontanesi, Piero Porcelli, Luca Tommasi

**Affiliations:** Department of Psychological, Health and Territorial Sciences, “G. d’Annunzio” University of Chieti-Pescara, Chieti, Italy

**Keywords:** COVID-19, anxiety, pre-traumatic stress reactions, protective behaviors, emotional worry

## Abstract

The World Health Organization defined COVID-19 as a pandemic on March 11, due to the spread of the new SARS-CoV-2 coronavirus in all continents. Italy had already witnessed a very fast spread that brought the Government to place the entire country under quarantine on March 11, reaching more than 30,700 fatalities in 2 months. We hypothesized that the pandemic and related compulsory quarantine would lead to an increase of anxiety state and protective behaviors to avoid infections. We aimed to investigate whether protective behaviors might have been enhanced or limited by anxiety and emotional reactions to previous experience of stressful conditions. We collected data from 618 Italian participants, by means of an online survey. Participants were asked to rate their level of worry for the pandemic, and to complete two questionnaires measuring the anxiety level: the state-trait anxiety inventory (STAI-Y) and the Pre-traumatic stress reaction checklist (Pre-Cl). Finally, the respondents were also asked to report about their compliance with protective behaviors suggested to avoid the spread of the virus (e.g., washing hands). Results show that respondents with higher levels of worry reported higher levels of anxiety and pre-traumatic reactions, with positive correlations among the three measurements, and that higher frequency of the three protective behaviors were put in place by respondents with higher levels of worry. Moreover, regression analysis showed that worry for COVID-19 was most predicted by age, anxiety levels, and Pre-traumatic stress. These results could be interpreted in an evolutionary framework, in which the level of worry leads persons to become more cautious (protective behaviors) maximizing long-term survival at the cost of short-term dysregulation (anxiety).

## Introduction

Beginning in the last months of 2019, a new coronavirus has spread worldwide triggering a viral pandemic in a few weeks, known as COVID-19, involving a respiratory syndrome with potentially severe complications (Cascella et al., 2020). This new coronavirus had been firstly isolated in Wuhan, China (Li et al., 2020b), but in a few weeks, the virus managed to infect the whole world being defined as a pandemic by the World Health Organization on March 11, 2020. This means that all of us were suddenly exposed to daily information about the dramatic impact of the epidemic on global health. The new term “infodemic” was coined and referred to the great amount of information available online and by means of traditional and social media which is not always truthful or controlled by reliable sources. The worldwide consequences of the pandemic have been and will continue to be highly dramatic in terms of social, financial, and individual burden such as mortality (356,000 deaths at the present time), morbidity (about two million persons infected in 5 months), deprivation of personal freedom due to the recommended or imposed quarantine, and about one trillion dollars that has been estimated to be lost^1^ (April, 2020). The cost in terms of psychological pressure has been heavy as well. It has been recently found, for instance, that healthy persons exposed to higher doses of media information about COVID-19 also reveal higher psychological distress (Yao, 2020).

In this scenario, Italy paid a very high price with more than 30,000 deaths from the end of February to mid-April, becoming the first and most afflicted country in Europe and in the world in that period. Preliminary epidemic data showed that male individuals had a higher likelihood to contract the virus compared to females (2/3 of the Italian infected patients were males) and, once infected, males were more likely to need hospitalization and to suffer from serious consequences than females (Onder et al., 2020). Moreover, COVID-19 was found to be more dangerous for older persons than for younger ones and for patients suffering from other chronic illnesses (Remuzzi and Remuzzi, 2020). A number of online studies proliferated worldwide with the aim to understand the impact of the epidemic on psychological variables such us depression and anxiety, as for instance, in China (Huang and Zhao, 2020; Lei et al., 2020; Li et al., 2020a), Iran (Moghanibashi-Mansourieh, 2020), Turkey (Özdin and Bayrak Özdin, 2020), Spain (Ozamiz-Etxebarria et al., 2020), and Italy (Mazza et al., 2020). All of these studies confirmed the psychological cost of the pandemic, compulsory quarantine, and excessive media exposure (infodemic).

### The Current Study

The general aim of this study was to investigate whether anxiety states and previous experiences of stressful conditions (pre-traumatic stress reactions) would influence the adoption of protective behaviors in order to avoid infection and to protect individual health (for a theoretical model see Freeston et al., 2020). In particular, we first investigated the possible effects of demographic differences on the anxiety level in the general Italian population. We hypothesized (1) that personal variables (gender, age, education, and occupation), as well as living in highly infected zones, could have an impact on the anxiety levels connected to COVID-19 infection. Then, because the quarantine period was made compulsory in Italy to all of the population since the 9th of March, we aimed at providing an overview of the daily protective habits of Italians, investigating the proclivity to adopt the behaviors suggested by the WHO (washing hands, opening windows, disinfecting living environments). Thus, we also hypothesized (2) that high levels of concern and worry for COVID-19 could have an impact on the protective behaviors (Brooks et al., 2020; Li et al., 2020c) by enhancing an abnormal illness behavior (Lipowski, 1987) toward fueling hypochondriacal concerns or avoid behavioral recommendations. In fact, recent studies limited to parents and families, suggest that anxiety levels are connected to safety behaviors, but the health risks and fear connected to COVID-19 influence the rates of stress (Lauri Korajlija and Jokic-Begic, 2020; Spinelli et al., 2020). Most of the studies concerning previous pandemics had focused on either the cognitive aspects related to what the population knew about the illness and what people really did to prevent the spread of the pandemic (Barr et al., 2008), or on the affective aspects of the disaster, investigating post-traumatic stress disorder (PTSD), depression, or anxiety (Goodwin et al., 2011; Karademas et al., 2013). Finally, we explored (3) whether anxiety states determined by the current situation and individual predisposition to anxiety reactions (pre-traumatic stress reactions) might facilitate or inhibit the suggested protective behaviors. To our knowledge, the relationship between anxiety and protective behaviors during the COVID-19 lockdown has received very little attention, with respect to other psychopathological domains. It might constitute an important helpful evidence to understand whether and up to which extent the suggested guidelines to prevent the contagion can be affected by the psychological states (namely anxiety and stress) and by demographical differences (e.g., age, gender, and regional areas). Importantly, as it would be difficult to identify people meeting DSM-5 (American Psychiatric Association, 2013) diagnostic criteria for PTSD, because the pandemic is still ongoing, participants were asked to complete a questionnaire already used with Afghanistan veterans to measure their pre-traumatic stress reactions namely the Pre-Cl scale. Previous studies have indeed shown that pre-traumatic stress reactions are a valid predictor of PTSD (Berntsen and Rubin, 2015).

## Materials and Methods

### Data Sources and Procedure

Between March 26 and April 8, we used an online link to invite Italian participants to take part in a survey on the effects of COVID-19. During these 2 weeks, COVID-19 epidemic showed a great spread in Italy. On March 26, 62,013 persons were recorded as newly infected and 8,165 died because of COVID-19; on April 8, infected people raised at 95,262 and deaths to 17,669^2^ on a total Italian population of 60,317,000 inhabitants. The survey was created and redistributed by using Qualtrics XM^3^. Participants completed the survey only after indicating their consent on a form that described the study aims, participant rights, and data treatment procedure. Participants were recruited through social media and snowball sampling. The survey took approximatively 20 min to complete, and participation was voluntary, anonymous, and free. Due to both the lack of previous similar data available when the online questionnaire was built and the need to obtain responses in a specific time window, the sample size was not specifically calculated *a priori.* At the beginning of the survey, the participants were informed that they would be asked to respond to a series of questions, specifying that all data would be treated anonymously and they were asked to agree with the informed content by clicking a button, otherwise they were redirected outside of the survey. The research was conducted in accordance with the ethical principles stated in the Declaration of Helsinki (World Medical Association, 2013), and approved by the Institutional Review Board of Psychology (IRBP) – Department of Psychological, Health and Territorial Sciences, Università degli Studi “G. d’Annunzio” Chieti-Pescara (id. nr. 20009).

The survey was composed of different sections. Here, we report data about socio-demographic information, anxiety level measured by using the STAI-Y questionnaire (Spielberger et al., 1983a), pre-traumatic stress reactions measured by using the Pre-Cl questionnaire (Berntsen and Rubin, 2015), affective worry (AW) measured by means of a list of questions adapted from a previous study (Liao et al., 2014), and protective behaviors constituted by three items about the daily behaviors recommended by the WHO in order to prevent the spread of COVID-19. When unavailable, the Italian translation was made *ad hoc* and validated by a bilingual person.

### Demographic Data

The survey was fully completed by 618 participants, including 441 females (71.36%) and 177 males (28.64%). The age of the sample ranged from 19 to 80 years old (means ± SE: 38.55 ± 0.61; SD = 15.26) and four age groups were created: group (a) 19–25 years old (*N* = 161, 26.01%); group (b) 26–35 years old (*N* = 164, 26.5%); group (c) 36–50 years old (*N* = 163, 21%); and group (d) 51–80 years old (*N* = 86, 26.4%). Education levels showed that 282 (45.7%) participants have a high school diploma (13 years of study), 235 (38%) have achieved the bachelor’s or master’s degree, and 101 (16.34%) have achieved a post-graduate degree. As regards with the current occupation, in our sample, 169 (23.35%) participants are students, 341 (55.18%) have a regular job, and 118 (17.47%) are retired or unemployed. These three classes were also grouped under two main categories: “unoccupied” (*N* = 265, 43%) and “occupied” (*N* = 353, 57%). Sixty-seven participants (10.8%) have declared to live in the so called “Redzones,” namely the Northern Italian regions with highest rates of deaths and infections (Lombardy, Veneto, Emilia-Romagna, and Piemonte) accounting for the 71.32% of all the COVID-19 cases in our Country.

### Measurements

#### Anxiety

The Italian version of the State-Trait Anxiety Inventory, STAI-Y1 (Spielberger et al., 1983b; Pedrabissi and Santinello, 1989) was used to measure the current level of anxiety. The questionnaire is composed of 20 items investigating the general feelings of respondents on a 1–4 Likert scale. Ten items are focused on negative feelings and 10 items are focused on positive feelings. Responses on the positive items were reversed, so that higher scores to the STAI correspond to a higher level of anxiety (range: from 1 to 80). The mean score of the whole sample was 48.92 (±0.42), Cronbach’s alpha for the present research is 0.94.

#### Pre-traumatic Stress Reactions

The Pre-traumatic stress reaction Check List (Pre-Cl, Berntsen and Rubin, 2015) is a 20-item questionnaire investigating the psychological reactions to dangerous events, which at the moment of administration are still active. It has been shown to significantly correlate with the measurement of PTSD, as already found with Danish soldiers employed in Afghanistan (Berntsen and Rubin, 2015), showing its potential as a possible tool to predict the stress-related reaction in the population involved in the pandemic without the need to wait for the emergence of a PTSD diagnosis. It investigates the feelings of respondents in the last month on a 0–4 Likert scale. The final score ranges from 0 to 80, with higher scores corresponding to higher pre-traumatic reactions (e.g., intrusive involuntary images of possible future stressful events and their associated avoidance and increased arousal). As proposed by the authors who elaborated the questionnaire, pre-traumatic stress reactions are defined as disturbing future-oriented cognitions and imaginations which can be part of PTSD investigated by a temporal reversal of the past-directed items used in the diagnosis of PTSD. The advantage of this measure is that it can quantify a “sub-component” of a possible PTSD, during–not after–the traumatic event. Pre-Cl was translated in Italian and the mean score of the sample was 26 (±0.66). It could be of interest to underlie that the mean Pre-Cl score measured in 211 soldiers was 22.85 (Berntsen and Rubin, 2015). For the present research, Cronbach’s alpha is 0.92.

#### Affective Worry

Affective worry represents the emotional response to the risk of being infected with COVID-19. The levels of apprehension and concern for contracting the new coronavirus was measured by five items adapted from a study investigating the 2009 influenza AH1N1 pandemic in Hong Kong (Liao et al., 2014), and specifically translated in Italian: a 7-point Likert scale was used for three items, measuring (i) the level of concern to have contracted the new coronavirus with respect to a “seasonal flu” in case of flu-like symptoms, (ii) the level of concern to contract the new coronavirus in the next 1 month, and (iii) the level of concern to contract the new coronavirus in the next 1 month with respect to the overall population. A 5-point Likert scale was used to measure the level of concern to have contracted in the past 1 week the new coronavirus. A 10-point Likert scale was used to investigate the current level of concern toward the new Coronavirus. In all of the items, higher scores correspond to a higher level of concern. Cronbach’s alpha for the present research is 0.75.

#### Protective Behaviors

The last part of the survey was aimed at quantifying the protective behavior acted by the respondents and corresponding to the recommendation suggested by the WHO in order to avoid the spread of the virus. In particular, participants were asked whether in the past 7 days they had (i) washed their hands more often than usual, (ii) cleaned and disinfected their house more often than usual, and (iii) often opened home windows to maintain good ventilation. Moreover, in case of a positive response, participants were asked to state whether that behavior was carried out specifically to prevent the infection spread or for other reasons. For these three items, the responses were coded as one if the respondents declared to have carried out the behavior to prevent the Coronavirus spread, otherwise they were coded as 0.

### Statistical Analysis

Data were analyzed using IBM SPSS 26. *T*-tests and analysis of variance (ANOVA) were used to analyze the differences between subgroups in the study variables. Cohen’s d was used as effect size index for the comparison between means and Tukey’s HSD for ANOVA *post hoc* analysis. Pearson correlation analysis was used to assess the correlation between the study variables. Hierarchical linear regression model was used to evaluate the influences of personal factors and psychological variables on the affective worries. Predictors were personal factors (gender, age, and occupation), COVID-19-related experiences (living in a high infected density area), and psychological variables (Pre-Cl and STAI-Y).

## Results

### Personal Variables and Anxiety

Table 1 shows the sociodemographic characteristics and scale scores of the sample. Female participants scored significantly higher than male participants to psychological scales of Pre-Cl (*d* = 0.60), STAI-Y (*d* = 0.67), and in the AW (*d* = 0.38). ANOVA *post hoc* results showed that Pre-Cl and STAI-Y scores (*p* < 0.05 and *p* < 0.01, respectively) were significantly higher in less educated participants and students (*p* < 0.01 and *p* < 0.01, respectively), which was to be expected because a large number of participants with a high school diploma were college students (35%). Surprisingly, living in a highly COVID-19 infected areas (*redzones*) did not affect the psychological scales scores. Pearson correlation analysis showed that trait anxiety (STAI-Y) was largely associated with Pre-Cl (*r* = 0.708, *p* < 0.01) and moderately with AW (*r* = 0.434, *p* < 0.01) that, in turn, was moderately associated with Pre-Cl (*r* = 0.397, *p* < 0.01) (data not shown; available at request to the corresponding author).

**TABLE 1:** Characteristics of the sample.

	Pre-Cl		STAI-Y		AW	
Demographic variables	*M*	DS	*M*	SD	*M*	SD
**Gender**						
*Men*	19.78	14.25	44.14	9.76	18.71	5.10
*Women*	28.78	15.68	50.84	10.06	20.64	5.00
*t*(p)	−6.89 (<0.001)		−7.55 (<0.001)		−4.31 (<0.001)	
*d*	0.60		0.67		0.38	
**Age**						
*(a) 19–25*	32.23	14.84	52.08	9.62	19.83	4.61
*(b) 26–35*	26.12	15.36	48.43	10.62	19.57	5.05
*(c) 36–50*	22.65	15.84	47.14	10.17	20.50	5.15
*(d) Over 51*	23.18	15.55	47.71	10.59	20.53	5.55
*F*(p)	12.66 (<0.001)		7.27 (<0.001)		1.39 (0.345)	
*Tukey’s HSD*	a>b,c,d		a>b,c,d			
**Education**						
*(a) High School diploma*	27.18	16.54	49.77	10.50	20.41	5.21
*(b) Bachelor/Master Degree*	26.86	15.09	49.21	10.10	19.87	5.07
*(c) Ph.D.*	21.95	14.78	45.89	10.45	19.68	4.88
*F*(p)	4.44 (0.012)		5.36 (0.015)		1.08 (0.340)	
*Tukey’s HSD*	c<a,b		c<a,b			
**Occupation**						
*(a) Student*	31.84	14.13	51.91	9.37	19.71	4.46
*(b) Worker*	24.39	16.38	47.61	10.82	20.43	5.28
*(c) Unoccupied*	23.93	14.30	48.84	9.68	19.51	5.34
*F(*p)	13.97 (<0.001)		9.49 (<0.001)		1.93 (0.15)	
*Tukey’s HSD*	a>b,c		a>b,c			
**Living in Redzones**						
*Yes*	25.84	15.48	47.38	10.75	20.13	4.48
*No*	26.22	15.84	49.01	10.40	20.09	5.14
*t*(p)	0.62 (0.53)		−0.03 (0.97)		1.24 (0.21)	
*d*	0.02		0.15		0.01	

### The Effects of Concern for COVID-19 on Protective Behaviors

Table 2 shows the characteristics of suggested protective behaviors. People who carry out protective behaviors due to concern about COVID-19 infection showed higher levels of AW. In particular, people who wash their hands more frequently due to the fear of being infected showed significantly higher levels of AW than other participants (*d* = 1.04), a moderate effect was also found in participants who disinfected or cleaned their house due to COVID-19 (*d* = 0.63). Participants who open their windows to refresh their house to prevent the infection of COVID-19 showed moderately higher levels of Pre-Cl (*d* = 0.26) and trait anxiety (*d* = 0.32), and higher levels of AW (*d* = 0.56). Table 3 shows the hierarchical regression model for predicting AW from sociodemographic and psychological variables. Being older (*B* = 0.04, *β* = 0.11, *p* < 0.01), and having an occupation during the lockdown (*B* = 1.50, *β* = 0.15, *p* < 0.001) were significantly associated to AW, even though they predicted only less than 1% of its variance. Trait anxiety (*B* = 0.15, *β* = 0.30, *p* < 0.001) and Pre-Cl (*B* = 0.70, *β* = 0.20, *p* < 0.001) showed higher association with AW by explaining 24% of its added variance.

**TABLE 2:** Means, standard deviation and differences in the study variables between protective behaviors groups.

	Pre-Cl		STAI-Y		AW	
	*M*	*SD*	*M*	*SD*	*M*	*SD*
**Wash hands**						
*COVID-19 (N* = *592)*	26.28	15.78	49.03	10.36	20.30	5.00
*Other (N* = *26)*	24.50	16.71	46.35	11.48	15.19	4.97
*t(p)*	0.532 (ns)		1.28 (ns)		1.28 (<0.001)	
*d*	0.11		0.24		1.02	
**Disinfected/clean the house**						
*COVID-19 (N* = *487)*	26.64	15.87	49.26	10.35	20.75	4.97
*Other (N* = *131)*	24.58	15.51	47.66	10.60	17.61	4.91
*t(p)*	1.32 (ns)		1.55 (ns)		6.44 (<0.001)	
*d*	0.13		0.15		0.63	
**Open windows**						
*COVID-19 (N* = *244)*	28.68	16.68	50.93	10.50	21.75	4.71
*Other (N* = *374)*	24.59	15.01	47.61	10.16	19.00	5.08
*t(p)*	3.17 (<0.001)		3.90 (<0.001)		6.78 (<0.001)	
*d*	0.26		0.32		0.56	

**TABLE 3:** Hierarchical regression analysis for personal and psychological variables predicting affective worry (AW).

	B	SE	β	*R*	*R*^2^
*Step 1*				0.20	0.04***
Gender	0.78	0.42	0.07		
Age	0.04	0.01	0.11**		
Education	−0.41	0.26	−0.06		
*Step 2*				0.23	0.05*
Redzones	0.45	0.58	0.03		
Occupation	1.50	0.40	0.15***		
*Step 3*				0.49	0.24***
STAI-Y1	0.15	0.02	0.30***		
Pre-Cl	0.07	0.02	0.20***		

The tabled values for beta reflect Bs after Step 3, *p < 0.05; **p < 0.01; ***p < 0.001. Gender: 1, male; 2, female; Redzones: 1, living in a Redzone; 0, not living in a Redzone; Occupation: 1, working, 0, student/not unoccupied.

## Discussion

A number of studies published in the last weeks (Liao et al., 2014; Asmundson and Taylor, 2020; Huang and Zhao, 2020; Lei et al., 2020; Mazza et al., 2020; Moghanibashi-Mansourieh, 2020; Ozamiz-Etxebarria et al., 2020; Özdin and Bayrak Özdin, 2020) showed increased anxiety and stress levels due to the COVID-19 pandemic and lockdown. The present study was aimed at investigating the behavioral impact of emotional responses to such a stressful event. As expected, the results of the present study confirm gender and age differences on psychological reactions to COVID-19 consistent with another recent Italian report (Mazza et al., 2020). Women and younger adults scored significantly higher to trait anxiety (STAI-Y), pre-traumatic stress levels (Pre-Cl), and AW than men and older participants. These results are in line with previous investigations showing overall higher levels of anxiety (McLean et al., 2011; Li and Graham, 2017) and vulnerability to experience post-traumatic reaction in women than in men (Sareen et al., 2013). Furthermore, younger adults are likely to be more exposed to “infodemia” because they can be more exposed to social media and the Internet (Siliquini et al., 2011) and, therefore, more vulnerable to increased anxiety and stress attributable to this massive and uncontrolled exposition to pandemic information (Yao, 2020). Another possible explanation for this latter result is that younger participants may have a lower psychological buffer because of a lower educational level since younger age, student status, and education all experienced more anxiety than the other subgroups. Surprisingly, living in a “redzone” (i.e., in a highly infected area with higher mortality rates) did not influence emotional reactions or behavioral habits. This may be due to the fact that less than 11% of our sample lived in a “redzone” or that the local impact of the infection were less powerful than the “infodemic” influence on psychological states. Overall, within our sample, only gender is related to the AW, whereas age, educational level, occupation, and gender are related to both anxiety and stress reactions. This pattern of results is partially confirmed by the regression model showing that 24% of the total variance of AW is explained by older age, having a job during the lockdown, and stress.

Our aim was also to assess the effect of these psychological traits on the daily behavior suggested in order to prevent the spread of the virus. To this aim, we took into account three specific behaviors (frequent hands washing, house disinfection, and opening windows), and asked participants whether they complied with these behaviors in the last weeks with the specific aim to prevent the pandemic. The results showed that hand washing and house cleaning/disinfecting are not influenced by either anxiety or stress levels, while participants with higher scores in both anxiety and stress scales are more prone to open the windows to ventilate the living environments. Furthermore, all of the three protective behaviors (hand washing, house disinfecting, and opening the windows) are influenced by the AW: participants with a higher level of worry about the COVID-19 declared to carry out each behavior more than the participants with a lower level of AW. The present data suggest that the anxiety connected to the fear of COVID-19 infection can be the motivation to engage in the recommended protective behaviors.

It is also relevant to note that our sample scores are relatively higher in the STAI-1Y scale. In fact, 63% of participants reported a score higher than 40, which researches suggest to be the clinical cut-off score for moderate symptoms, and the 14% scored higher than 60, which is the cut-off score for severe clinical anxiety symptoms (Pedrabissi and Santinello, 1989; Barisone et al., 2004). It is possible to suggest that, in line with other studies (Marchetti et al., 2020; Mazza et al., 2020), the general population’s levels of anxiety and stress symptoms have risen due to COVID-19 fear and uncertainty.

A final remark has to be made concerning the measured stress. As specified, we measured the stress level by means of the Pre-Cl, a scale previously used with Afghanistan soldiers before, during, and after their war experience (Berntsen and Rubin, 2015). This scale has been shown to significantly predict the PTSD symptoms in that population, and we used this scale in order to have a rapid frame of a possible PTSD in the general population, at least in Italy, once the medical emergency will be controlled (namely, after the traumatic period). These results may be intended as a snapshot of a possible escalation of PTSD in the world, although caution is needed about the possibility to generalize this conclusion. In fact, it should be highlighted that an online survey was the only tool available to collect data during the quarantine. Nevertheless, due to the specific methodology used, one of the limitations is the uncontrolled representativeness of the sample (e.g., higher proportion of younger than older respondents, as well as of women rather than men). Similarly, some of the psychological scales used in the present study are not specifically validated for the online testing, and in particular, the Pre-Cl is a scarcely used test, which has been employed with Danish soldiers and it was not used in circumstances similar to those here described. For the same reason, even if it has been shown that in the military sample Pre-Cl scores significantly correlate with PTSD (Berntsen and Rubin, 2015), we could hypothesize a generalization of such a correlation to the sample tested here, but further studies are needed in order to confirm this possibility. Finally, due to the impossibility to assess a previous diagnosis of anxiety and related disorder [e.g., obsessive compulsive disorder (OCD)], our results, while promising, can be subjected to two biases. Firstly, we investigated some behaviors that are salient for people with OCD, and this could have had an impact on some participants’ answers. Then, the participants with previous diagnosis of anxiety disorders may have found themselves in an uncomfortable situation while participating in the survey, and that may have raised the levels of anxiety. Due to these reasons, future research should investigate the effect of lockdown and COVID-19 related behavior specifically in clinical samples.

We can conclude that targeted interventions by governments and institutions in support of the psychological wellbeing of the general population are desirable. The present results suggest that a particular attention should be focused on the part of population who had shown to be more prone to anxiety and stress, namely women, younger people, and students, who could be exposed to a real post-traumatic stress disorder.

## Data Availability Statement

The raw data supporting the conclusions of this article will be made available by the authors, without undue reservation.

## Ethics Statement

The study was designed and carried out in accordance with the World Medical Association Declaration of Helsinki and its subsequent revisions, and approved by the Institutional Review Board of Psychology – Department Of Psychological, Health And Territorial Sciences, University “G. d’Annunzio” of Chieti-Pescara. The participants provided their online informed consent to participate in this study.

## Author Contributions

GP and LT: conceptualization. GP, LF, and LT: methodology and investigation. LF and PP: formal analyses. GP and LF: writing – original draft. PP and LT: writing – review. All authors contributed to the article and approved the submitted version.

## Conflict of Interest

The authors declare that the research was conducted in the absence of any commercial or financial relationships that could be construed as a potential conflict of interest.

## Footnotes

1 https://unctad.org/en/Pages/Home.aspx

2 http://opendatadpc.maps.arcgis.com/apps/opsdashboard/index.html#/b0c68bce2cce478eaac82fe38d4138b1

3 https://www.qualtrics.com/it/?rid=langMatch&prevsite=en&newsite=it&geo=IT&geomatch=
